# Phosphatase of regenerating liver-3 directly interacts with integrin β1 and regulates its phosphorylation at tyrosine 783

**DOI:** 10.1186/1471-2091-13-22

**Published:** 2012-10-23

**Authors:** Wei Tian, Like Qu, Lin Meng, Caiyun Liu, Jian Wu, Chengchao Shou

**Affiliations:** 1 Key Laboratory of Carcinogenesis and Translational Research (Ministry of Education), Department of Biochemistry and Molecular Biology, Peking University Cancer Hospital & Institute, Beijing, China; 2 Department of Biochemistry and Molecular Biology, Peking University Cancer Hospital & Institute, 52 Fucheng Road Haidian District, Beijing 100142, China

**Keywords:** PRL-3, tyrosine phosphatase, integrin β1, dephosphorylation

## Abstract

**Background:**

Phosphatase of regenerating liver-3 (PRL-3 or PTP4A3) has been implicated in controlling cancer cell proliferation, motility, metastasis, and angiogenesis. Deregulated expression of PRL-3 is highly correlated with cancer progression and predicts poor survival. Although PRL-3 was categorized as a tyrosine phosphatase, its cellular substrates remain largely unknown.

**Results:**

We demonstrated that PRL-3 interacts with integrin β1 in cancer cells. Recombinant PRL-3 associates with the intracellular domain of integrin β1 *in vitro*. Silencing of integrin α1 enhances PRL-3-integrin β1 interaction. Furthermore, PRL-3 diminishes tyrosine phosphorylation of integrin β1 *in vitro* and *in vivo*. With site-specific anti-phosphotyrosine antibodies against residues in the intracellular domain of integrin β1, tyrosine-783, but not tyrosine-795, is shown to be dephosphorylated by PRL-3 in a catalytic activity-dependant manner. Phosphorylation of Y783 is potentiated by ablation of PRL-3 or by treatment with a chemical inhibitor of PRL-3. Conversely, depletion of integrin α1 decreases the phosphorylation of this site.

**Conclusions:**

Our results revealed a direct interaction between PRL-3 and integrin β1 and characterized Y783 of integrin β1 as a bona fide substrate of PRL-3, which is negatively regulated by integrin α1.

## Background

PRL-3, a non-receptor tyrosine phosphatase containing a CAAX motif for prenylation at the carboxyl terminus [1], was found to be up-regulated in various types of malignancy, including colorectal, gastric, ovarian and breast cancers [2-5]. It has been shown to play a causal role in tumor metastasis by enhancing cancer cell motility and in tumor angiogenesis by recruiting endothelial cells [2]. PRL-3 expression is correlated with disease progression and poor survival [3-8], and its antibody was shown to dramatically inhibit metastatic tumor formation in human ovarian cancer cells [9], therefore it had been deemed as a potential therapeutic target for the treatment of cancer [10].

PRL-3 could down-regulate PTEN expression and activate the PI3K pathway to promote epithelial-mesenchymal transition (EMT), thus contributing to tumor metastasis [11]. It has been implicated in controlling integrin-Src signaling pathway, in which ectopic PRL-3 promoted Src activation and potentiated Src-modulated oncogenic pathways, including ERK1/2, STAT3, and p130Cas [12]. PRL-3 was also shown to be a negative regulator of tumor suppressor p53 [13]. As a phosphatase, few PRL-3 substrates had been characterized, including Ezrin [14] and cytokeratin 8 [15], however, the cellular substrates of PRL-3 remain largely unknown.

Integrins are a large family of trans-membrane proteins, which are broadly involved in regulation of cell adhesion, motility and other physical and pathological processes [16,17]. Engagement of intergrins with their ligands stimulates diverse intracellular signaling pathways, such as tyrosine phosphorylation and activation of mitogen-activated protein kinases [18,19]. Integrin α1 and β1 are known to heterodimerize to form the receptor for extracellular matrix (ECM), which is prerequisite for downstream signaling [17]. We previously identified integrin α1 as an interacting protein of PRL-3 through yeast two-hybrid screening, and PRL-3 could down-regulate the tyrosine-phosphorylation level of integrin β1 [20]. Furthermore, we showed the critical role of integrin β1-ERK1/2-MMP2 signaling in PRL-3-promoted motility, invasion, and metastasis of colon cancer cells [21]. In this study, we demonstrate that PRL-3 directly binds to integrin β1 and dephosphorylates integrin β1-Y783, a key residue for integrin β1 function [22]. Moreover, we show that integrin α1 inhibits the PRL-3/integrin β1 interaction and the dephosphorylation of integrin β1-Y783 by PRL-3.

## Results

### Direct interaction between PRL-3 and integrin β1, which is regulated by integrin α1

**Figure 1:**
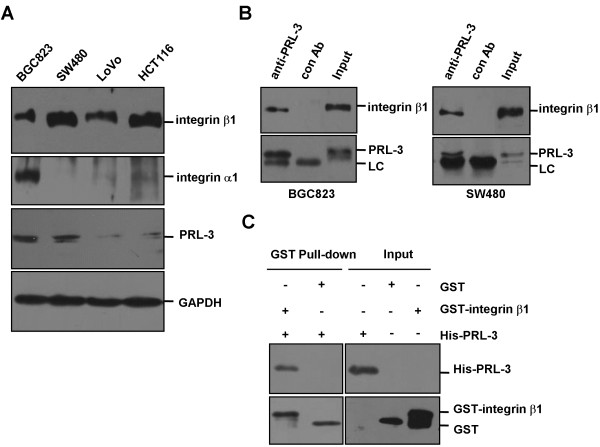
**Physical interaction between PRL-3 and integrin β1.** (**A**) The expression profiles of integrin β1, integrin α1 and PRL-3 in different cell lines. Equal amount of protein lysates from indicated cells were subjected to Western blotting with integrin β1, integrin α1and PRL-3 antibodies. GAPDH protein expression was shown as loading control. (**B**) Endogenous interaction between PRL-3 and integrin β1. 500 μg of lysates from BGC823 (left panel) or SW480 (right panel) cells were immunoprecipitated with 1 μg PRL-3 monoclonal antibody or 1 μg pre-immune mouse IgG (control), followed by Western blotting with integrin β1 and PRL-3 antibodies. Expression of integrin β1 and PRL-3 in the lysates (50 μg) was shown as input. (**C**) Direct interaction between PRL-3 and integrin β1. GST-integrin β1 (intracellular domain, 752-798aa) and His-tagged PRL-3 were expressed respectively and used for in vitro pull-down assay.

Our previous findings of integrin α1 as a binding partner of PRL-3 [20] and integrin β1-ERK1/2-MMP2 signaling mediating PRL-3-promoted cell invasion and metastasis [21] prompted us to further dissect the relationship between PRL-3 and integrins. As an initial step, we examined their expression levels in human gastric cancer cell line BGC823 and colon cancer cell lines SW480, LoVo, and HCT116. Figure 1A shows that integrin β1 was expressed in all these cell lines, but integrin α1 was only detected in BGC823 cells. In addition, relatively high expression of PRL-3 was found in BGC823 and SW480 cells (Figure 1A). Next, co-immunoprecititation assay was performed with protein lysates from BGC823 cells and SW480 cells. In both cell lines, endogenous integrin β1 was detected in anti-PRL-3 precipitates (Figure 1B). Furthermore, GST pull-down assay was performed with bacterially expressed His-tagged PRL-3 and GST-fused intracellular domain of integrin β1 (752-798aa). The result shows that His-PRL-3 could be precipitated by GST-intergrin β1, but not by GST (Figure 1C). Thus, PRL-3 directly interacts with integrin β1.

**Figure 2:**
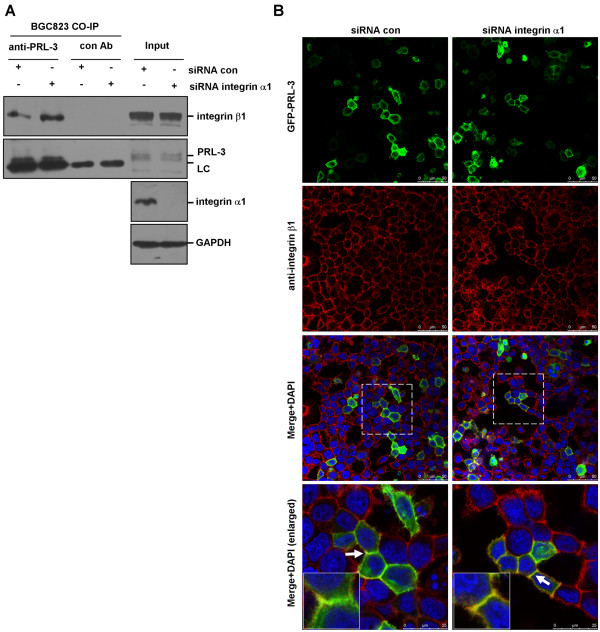
**Physical interaction between PRL-3 and integrin β1.** (**A**) Deletion of integrin α1 resulted in enhanced PRL-3-integrin β1 interaction. BGC823 cells were transfected with 100 pmol integrin α1-specific siRNA or control siRNA for 48 hr. 500 μg protein lysates were incubated with PRL-3 antibody or pre-immune mouse IgG (control) for co-immunoprecipitation assay. The immunoprecipitates and 50 μg protein lysates (input) were immunoblotted by integrin β1 and PRL-3 antibodies. Efficiency of intergrin α1 silencing in the input was verified. “LC” is short for light chain of antibody. (**B**) PRL-3 co-localized with integrin β1 in an integrin α1-independent manner. After being transfected with 100 pmol integrin α1 siRNA and control siRNA for 24 hours, BGC823 cells were transfected with 2 μg GFP-PRL-3, and cultured for another 24 hours, then fixed, blocked, and immunostained with anti-integrin β1 antibody. Localization of GFP-PRL-3 (green) and integrin β1 (red) was detected by a laser confocal microscope. Parts of merged images were enlarged (white rectangles) to show the co-localization between two molecules (yellow). The white arrows labeled regions were further enlarged (insert).

Since integrin α1 heterodimerizes with integrin β1 [16] and our previous study revealed an integrin α1-PRL-3 interaction [20], we then investigated whether integrin α1 plays a role in modulating PRL-3-integrin β1 interaction. We transfected integrin α1-specific siRNA or control siRNA into integrin α1-proficient BGC823 cells, and performed co-immunoprecipitation assay. As shown in Figure 2A, more integrin β1 was precipitated by anti-PRL-3 antibody upon integrin α1-silencing. In addition, we carried out immunoflorescence assay to examine the co-localization between GFP-PRL-3 and endogenous integrin β1. GFP-PRL-3 and integrin β1 partially co-localized on the punctate structures of cell membrane in control siRNA-transfected cells. However, knockdown of integrin α1 resulted in an increase in the co-localization between GFP-PRL-3 and integrin β1 (Figure 2B). These findings suggest that integrin α1 negatively regulates association between PRL-3 and integrin β1.

### *In vitro* and *in vivo* dephosphorylation of integrin β1 by PRL-3

**Figure 3:**
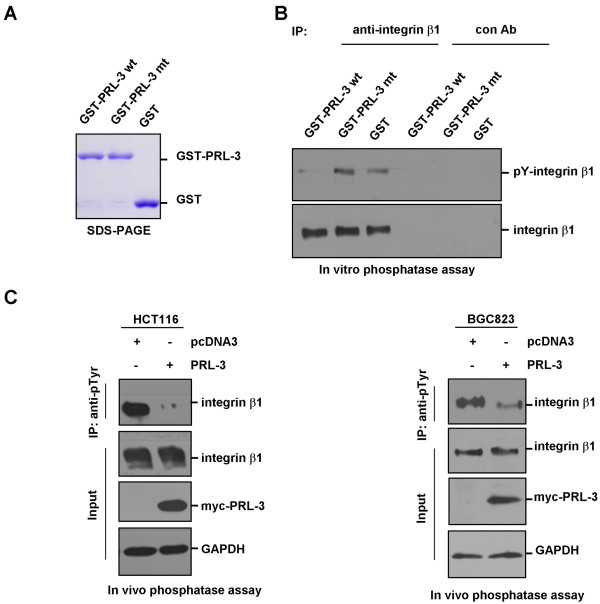
***In vitro *and *in vivo *dephosphorylation of integrin β1 by PRL-3.** (**A**) The purified GST-PRL-3, mutant GST-PRL-3 (GST-PRL-3-mt) and GST proteins were examined by SDS-PAGE and commassie blue staining. (**B**) Dephosphorylation of integrin β1 by PRL-3 *in vitro*. 1000 μg HCT116 cell lysates were immunoprecipitated with integrin β1 antibody or control IgG. After sequential washing with lysis buffer and dephosphorylation buffer, the precipitates were used as substrates, and incubated with 1 μg purified GST-PRL-3 wt, GST-PRL-3 mt or GST in the dephosphorylation buffer for 30 min at 30°C, then the mixtures were analyzed by Western blotting with anti-phosphotyrosine (4G10) and anti-integrin β1 antibodies. (**C**) Dephosphorylation of integrin β1 in HCT116 (left panel) and BGC823 (right panel) cells by ectopic expression of PRL-3. Equal amount of lysates from cells over-expressing myc-PRL-3 and pcDNA3 (control) were immunoprecipitated with anti-phosphotyrosine (4G10) antibody, followed by immunoblotting with anti-integrin β1 antibody. Expression of integrin β1 and myc-PRL-3 in the lysates were indicated.

Since PRL-3 directly interacts with integrin β1, we further investigated if integrin β1 is a substrate of PRL-3 phosphatase. We expressed and purified GST-fused wild-type PRL-3 and the PRL-3-mt, in which the phosphatase activity was eliminated by mutating cystine 104 to serine [12,23] (Figure 3A). *In vitro* dephosphorylation assay was performed with GST-PRL-3 and GST-PRL-3-mt plus equal amount of immunoprecipitated endogenous integrin β1 as substrate. We found wild-type GST-PRL-3 significantly decreased the tyrosine phosphorylation of integrin β1, whereas mutant GST-PRL-3 had no obvious effect (Figure 3B).

Moreover, we examined if such regulation occurs *in vivo*. We introduced myc-PRL-3 into BGC823 and HCT116 cells. As controls, the cells were transfected with vector alone. Immunoblot analysis revealed that ectopic expression of myc-PRL-3 considerably decreased the phosphorylation of integrin β1 at tyrosine residue(s), but did not affect total protein levels of integrin β1 (Figure 3C). These results indicate that PRL-3 dephosphorylates integrin β1 *in vitro* and *in vivo*.

### PRL-3 dephosphorylates tyrosine-783 of integrin β1

**Figure 4:**
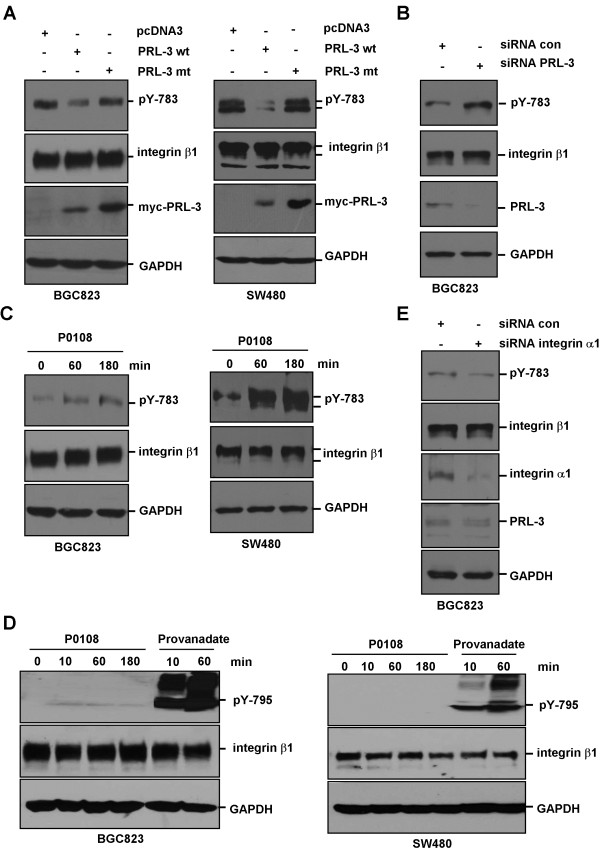
**Regulation of integrin β1 phosphorylation at tyrosine 783 by PRL-3.** (**A**) Wild-type myc-PRL-3, but not mutant myc-PRL-3 dephosphorylated tyrosine 783 of integrin β1 in BGC-823 (left panel) and SW480 cells (right panel). After transfection with myc-PRL-3, myc-PRL-3-mt or pcDNA3 (control) plasmids, equal amount of cell lysates were analyzed by Western blotting with antibodies against pY783, integrin β1, myc and GAPDH. (**B**) Knockdown of PRL-3 promoted pY783 phosphorylation. BGC823 cells were transfected with 100 pmol indicated siRNA for 48 hours, and the cell lysates were analyzed by Western blotting with indicated antibodies. (**C**) PRL-3 inhibitor enhanced pY783 phosphorylation. BGC823 (left panel) and SW480 cells (right panel) were treated with 10 μg/ml PRL-3 inhibitor. Cells were harvested at different time points and protein lysates were analyzed by Western blotting with indicated antibodies. (**D**) PRL-3 had minimal effect on pY795 of integrin β1. BGC823 (left panel) and SW480 cells (right panel) were treated with 10 μg/ml PRL-3 inhibitor or 100 μM provanadate (as positive control). Protein lysates were harvested at indicated time points and analyzed by Western blotting. (**E**) Knockdown of integrin α1 decreased pY783 phosphorylation. BGC823 cells were transfected with integrin α1-specific siRNA or control siRNA for 72 hours, and the lysates were analyzed by Western blotting.

Previous study reported that phosphorylation of Y783 and/or Y795 in the cytoplasmic domain of integrin β1 may be essential for its function [24]. We next investigated whether PRL-3 dephsohorylates Y783 and/or Y795. BGC823 cells and SW480 cells were transfected with myc-tagged wild-type PRL-3 and PRL-3-mt. Immunoblot analysis revealed that ectopic expression of PRL-3, but not PRL-3-mt, significantly reduced pY783 of integrin β1 in both cell lines (Figure 4A), while pY795 were undetectable (data not shown). We also knocked down endogenous PRL-3 and found that depletion of PRL-3 could markedly increase pY783 levels in BGC823 cells (Figure 4B). To further validate PRL-3-mediated dephosphorylation of Y783 of integrin β1, chemical inhibitor of PRL-3 (P0108), which has been shown to specifically inhibit PRL-3 phosphatase activity and decrease PRL-3-mediated cancer cell migration and invasion [15,25], was used to treat BGC823 and SW480 cells. Increment of pY783 levels in both cell lines was observed after P0108 treatment (Figure 4C). P0108 had a marginal stimulatory effect on pY795 in BGC823 cells, but pY795 could be strongly enhanced by treatment with tyrosine phosphatase inhibitor pervanadate in both BGC823 and SW480 cells (Figure 4D). In line with enhanced PRL-3-integrin β1 interaction upon silencing of integrin α1 (Figure 2A and B), pY783 was decreased by ablation of integrin α1 (Figure 4E). Collectively, these results show that PRL-3 dephosphorylates integrin β1 at tyrosine-783, but not tyrosine-795.

## Discussion

Protein kinases and phosphatases play important roles in diverse physiological processes and diseases [26-28]. PRL-3, as a member of protein phosphatases, has been found to promote cancer cell invasiveness [10,29-32]. However, the underlying mechanism remains elusive. In the present study, we demonstrate that PRL-3 directly binds to integrin β1 and dephosphorylates integrin β1-Y783. Furthermore, we found that deletion of integrin α1 resulted in increased PRL-3-integrin β1 association and decreased phosphorylation of integrin β1-Y783. These findings indicate that integrin β1 is a bona fide substrate of PRL-3 and raise the possibility that integrin α1 may function as a negative regulator for PRL-3-integrin β1 interaction.

Previous studies have shown that integrin β1-Y783 and -Y795 are parts of conserved NPxY motifs essential for recruiting talin and kindlin, which in turn facilitates coupling of integrin β1 to the actin cytoskeleton and maintains integrins in an active signaling state [33]. It was reported that v-Src could phosphorylate integrin β1 tails on Y783 and Y795 [34,35], and that phosphorylated Y783 and Y795 could block talin and kindlin’s binding with integrin β1, respectively [36,37]. These were supported by the study that v-Src expression in fibroblasts decreases integrin β1–dependent adhesion, focal adhesion formation, cytoskeletal organization, fibronectin assembly, migration, and chemotaxis [34,38]. While several kinases, including Src, have been shown to phosphorylate Y783 and Y795 [34,35], the phosphatase(s) catalyzing the de-phosphorylation of these two sites is unknown. By showing PRL-3 is responsible for dephosphorylating pY783, our present results provide an explanation for the delicate control of integrin β1.

It is noted that a recent study showing the short phosphotyrosine peptides encompassing Y783 or Y795 of integrin β1 could not be dephosphorylated by PRL-3 in an *in vitro* assay [39]. However, as the author suggested, this could be explained by lack of entire integrin β1 to be recognized by PRL-3 [39]. In our study, instead of using the synthesized peptides, we immunoprecipited the endogenous integrin β1 as substrate for *in vitro* phosphatase assay, which ensures optimal phosphatase-substrate association. In addition, we did not find alteration in pY795 by over-expression or ablation of PRL-3, which could be due to the fact that pY795 level is too low to be detected in the cancer cells examined. We did observe slightly more pY795 in PRL-3 inhibitor treated BGC823 cells, while such agent-induced changes of pY795 was not as robust as those of pY783 in BGC823 and SW480 cells. Interestingly, treatment with a pan-phosphatase inhibitor significantly elevates pY795, suggesting that PRL-3 may partially contribute to the dephosphorylation of pY795 and pY795 is mainly regulated by other phosphatase(s).

## Conclusion

In summary, our results demonstrated a direct interaction between PRL-3 and integrin β1, which could be negatively regulated by integrin α1. Importantly, we identified tyrosine 783 of integrin β1 as a direct dephosphorylation site by PRL-3, thus uncovering the first tyrosine phosphorylation site to be regulated by PRL-3 phosphatase.

## Methods

### Cell lines and Reagents

Colon cancer cell lines LoVo, SW480 and HCT116 were obtained from ATCC (Manassas, VA) and maintained in DMEM (Invitrogen, Carlsbad, CA). Gastric cancer cell BGC823 was maintained in RPMI-1640 medium (Invitrogen). The medium was supplemented with 10% fetal calf serum (Invitrogen). The antibodies against integrin β1 (MAB 2000), integrin α1 (MAB1973) and phosphorylated tyrosine (4G10) (16–316) were from Millipore (Billerica, MA). Anti-phosphorylated integrin β1 (Tyr783) (600601) and (Tyr795) (600501) antibodies were obtained from Biolegend (San Diego, CA). Antibody against PRL-3 (clone 318) was from Santa Cruz (Santa Cruz, CA). GADPH antibody was from Proteintech Group (Chicago, IL). PRL-3 inhibitor 1-(2-bromobenzyloxy)-4-bromo-2-benzylidene rhodanine (P0108) was from Sigma (St. Louis, MO). The tyrosine phosphatase inhibitor (Pervanadate) was freshly prepared by dissolving sodium orthovanadate (Sigma) with PBS to 30 mM, adding hydrogen peroxide at 0.18% (v/v), and incubating for 15 min at room temperature avoiding light before treating the cells at the final concentration of 100 μM.

### Plasmids transfection and RNA interference

The plasmids expressing myc-PRL-3, myc-PRL-3-mt (cystine 104 was mutated to serine) and GFP-PRL-3 were described as previously [23]. The small interference RNAs (siRNAs) targeting integrin α1 and PRL-3 were synthesized by GenePharma (Shanghai, China), the sequence for integrin α1: sense, 5’- GCCCUUAUAUGCCUAUAGA -3’; antisense, 5’- UCUAUAGGCAUAUAAGGGC -3’; the sequence for PRL-3: sense, 5’- CAGCAAGCAGCUCACCUAC -3’; antisense, 5’- GUAGGUGAGCUGCUUGCUG -3’. Plasmids and siRNA were transfected into cells with Lipofectamine 2000 (Invitrogen) following provider’s instruction.

### Immunofluorescence

To visualize the localization of integrin β1, BGC823 cells were cultured on the coverslips and fixed with 2% paraformaldehyde for 30 min at 4°C, followed by permeabilization with 0.5% Triton X-100 in PBS for 5 min, and blocking with 3% bovine serum albumin overnight at 4°C. After incubation with anti-integrin β1 (1: 300) for 1 hr at room temperature, cells were probed with tetramethyl rhodamine isothiocyanate-conjugated secondary antibody, counterstained with 4', 6-diamidino-2-phenylindole (DAPI), and mounted on 50% glycerol/PBS. Localization of PRL-3 was analyzed by transfecting the cells with pEGFP-PRL-3. A Leica SP2 confocal system (Leica Microsystems, Dresden, Germany) was used to observe the localization of integrin β1 and GFP-PRL-3.

### Western blotting and immunoprecipitation

For Western blotting, cells were directly lysed in 1x loading buffer. For immunoprecipitation assay, cells were homogenized in lysis buffer (50 mM HEPES pH 8.0, 150 mM NaCl, 1 mM EDTA, 1% Triton X-100, 10% glycerol, 50 mM NaF, 1 mM Na_3_VO_4_, 2 mM dithiothreitol, 1 × protease cocktail (Sigma)) for 20 min at 4°C. The supernatant was collected after centrifugation at 12,000 × g for 20 min at 4°C and then incubated with indicated antibodies conjugated to protein G-Sepharose (Invitrogen). Cell lysates or immunoprecipitates were separated by SDS-PAGE and electro-blotted to the nitrocellulose membranes. Non-specific binding was blocked with 5% non-fat milk in PBS overnight at 4°C and was rinsed twice with PBST. Then the membranes were incubated with indicated primary antibodies at room temperature for 1.5 h, and washed six times with PBST, followed by horseradish peroxidase-labeled secondary antibodies for 45 min and washed again as above. Protein bands were visualized with enhanced chemoluminescence system (Thermo Scientific, Rockford, IL).

### GST Pull-down assay

Deoxyribonucleic acids encoding intracellular domain of integrin β1 (752-798aa) was amplified by PCR with the following primers: sense, 5’- GACTGAATTCAAGCTTTTAATGATAATTCATG -3’; antisense, 5’- AGCAACTCGAGGTGTTGTGGGATTTGCAC -3’. The PCR product was digested by EcoR I/Xho I and inserted into pGEX-4T1 vector. His-tagged PRL-3 was constructed by inserting the digested PRL-3 into pET-28a vector. For pull-down assay, glutathione-Sepharose beads (Invitrogen) were incubated with E. coli bacteria lysates expressing GST-integrin β1 or GST. After being washed with PBS, these beads were incubated with His-PRL-3 in binding buffer (10 mM Tris–HCl pH 8.0, 150 mM NaCl, 1 mM dithiothreitol, 1 mM PMSF, 1% Triton X-100, 10% glycerol) for 4 hours at 4°C, then washed three times, boiled in SDS loading buffer, separated by SDS-PAGE and immunoblotted with PRL-3 and GST antibodies.

### *In vitro* phosphatase assay

Integrin β1 protein was immunoprecipitated from 1000 μg protein from HCT116 cell lysates with 1 μg integrin β1 antibody conjugated to protein G-Sepharose. For control, cell lysates were immunoprecipitated with 1 μg pre-immune IgG. After being washed 3 times with lysis buffer, once with dephosphorylation buffer (50 mM Tris pH 7.5, 2 mM dithiothreitol, 1 mM MgCl_2,_ 0.1 mM MnCl_2_), the precipitates were used as substrate for *in vitro* phosphatase assay. GST-PRL-3, GST-PRL-3-mt, and GST (1 μg each) were used as phosphatase and incubated with substrate in 20 μL dephosphorylation buffer at 30°C for 30 min. The reaction was stopped by boiling in 2 × loading buffer and the phosphorylated integrin β1 was detected by immunoblotting with 4G10 antibody.

## Abbreviations

PRL-3: Phosphatase of regenerating liver-3; PBS: Phosphate-buffered saline; SDS-PAGE: Sodium dodecyl sulfate polyacrylamide gel electrophoresis; siRNA: Small interference RNA; EMT: Epithelial-mesenchymal transition.

## Competing interests

The authors declare that there are not any financial or non-financial competing interests associated with this work.

## Authors' contributions

CS conceived the project. CS and LQ designed the experiments. WT, LM, CL, and JW performed the experiments. CS, LQ, and WT analyzed data. WT and LQ wrote the manuscript. All authors read and approved the final manuscript.
